# The Enemies of the Hospitals

**Published:** 1901-06-22

**Authors:** 


### The Institutional Workshop.

### THE ENEMIES OF THE HOSPITALS.

Hospital Sunday has come and gone, and we shall shortly see what has been the result of this great annual appeal to the charity of Londoners to help the sick and suffering in their midst. We cannot, however, let the occasion go by without raising a protest against the outrage committed in the neighbourhood of certain churches by the open distribution of handbills the object, and certainly the tendency, of which was to destroy the harmony of a great and otherwise unanimous effort in well-doing and to turn aside from the Hospital Sunday Fund the contributions of the charitable. It appears that the Hon. Stephen Coleridge addressed a letter to the incumbents of the churches in which he repeated his old misstatements as to the moneys of the Hospital Sunday Fund being "diverted" from their proper purpose, namely, "the relief of the sick poor," to the support of institutions wherein animals are vivisected, and urging them "as a matter of honour" to explain to their congregations that their contributions were liable to be so "diverted in part to these entirely different objects." This was followed up by the public distribution of a reprint of this letter, together with an advertisement of Mr. Coleridge's recently published "Guide to the Charitable," issued by the Anti-Vivisection Society. It was a despicable action, worthy only of the people who did it, people whose boasted humanity is of such a kind that they do not hesitate to sacrifice the interests of the sick poor to the furtherance of their own wretched fads.

This question of so-called vivisection and its connection with the hospitals has to be looked squarely in the face. There are certain things which can only be done, or can only be found out, by trial upon animals. Is this case tuberculosis? Is this diphtheria? Can we operate in this case or must the patient be left to die? these are questions which in many cases can only be answered by an appeal to an experiment on an animal—say a mouse or a guinea-pig. A rich man does not hesitate for a moment. He says this question must be decided; or, if it is a case of using an antitoxin for the cure of a disease, he says this thing must be done. And it is done. Why, then, for the sake of Mr. Coleridge and his fads are we to stand aside and deny to the poor man in hospital what without doubt the rich man would get at once? This is the only reasonable way of looking at this cruel attempt to draw away the charity of London from its great hospitals.

The Anti-Vivisection Society cares naught for hospitals or for the sick, and the whole bottom of these repeated attacks on the hospitals is that the Society wants to make life uncomfortable to certain men who are trying to do their best for their patients, even at the expense of a few mice and guinea-pigs. But we hope that the generous London public will take care to show that they care naught for Mr. Coleridge and his Society, and that they are determined that the sick poor in hospitals shall have the same advantages as are open to the rich outside. As to Mr. Coleridge's advertisement of his precious "Guide to the Charitable" this fact requires to be stated with the greatest clearness, namely, that the list which it contains of "hospitals that are now entirely free from any connection with vivisection," a list given for the guidance of intending benefactors, excludes every one of what may be properly called the great hospitals of London. It is nonsense to talk about this being a guide. It is a veiled, but for all that a perfectly definite, attempt to starve the great hospitals of London, and that for one sole reason—namely, that they offend Mr. Coleridge in their way of doing good. Fortunately the animus of the attack is sufficiently obvious, and fortunately also the reliability of the statements therein contained may be judged by internal evidence, for Mr. Coleridge includes among the "hospitals that are now entirely free from any connection with vivisection" a whole string of fever hospitals, hospitals which are daily engaged in the treatment of diphtheria with antitoxin, saving lives right and left by a proceeding which not only involves the vivisection of horses for the production of the antitoxin, but involves also a large amount of experimental vivisection for the standardisation of the product. So much for his accuracy. The whole affair is one of straining at gnats and swallowing camels. Vivisection is a thing from some connection with which no up-to-date hospital can cut itself entirely free. It is a proceeding which to ourselves personally is intensely distasteful, but it is one of those things which have to be done if the full benefit of existing knowledge is to be given to the sick. Moreover it is perfectly legal, if done under Government inspection, and under the special sanction of an Act of Parliament. This, however, is another matter. The point we stand to is that if vivisection is to be abolished it should be abolished for all alike. Let rich and poor stand in the same boat. What we think is scandalous is that Mr. Coleridge and his Society should attempt to enforce a difference, and that, with cruel indifference to the fate of the sick poor, he should endeavour to divert the gifts of the charitable from the Hospital Sunday Fund in order that he may give prominence to his own views regarding vivisection.

